# Proteomic and Carbonylation Profile Analysis at the Critical Node of Seed Ageing in *Oryza sativa*

**DOI:** 10.1038/srep40611

**Published:** 2017-01-17

**Authors:** Guangkun Yin, Xia Xin, Shenzao Fu, Mengni An, Shuhua Wu, Xiaoling Chen, Jinmei Zhang, Juanjuan He, James Whelan, Xinxiong Lu

**Affiliations:** 1 National Genebank, Institute of Crop Science, Chinese Academy of Agricultural Sciences, Beijing 100081, China; 2 China National Rice Research Institute, Hangzhou 310006, China; 3 Australian Research Council Centre of Excellence in Plant Energy Biology, School of Life Science, La Trobe University, Bundoora, Victoria 3083, Australia

## Abstract

The critical node (CN), which is the transition from the plateau phase to the rapid decreasing phase of seed ageing, is extremely important for seed conservation. Although numerous studies have investigated the oxidative stress during seed ageing, information on the changes in protein abundance at the CN is limited. In this study, we aimed to investigate the abundance and carbonylation patterns of proteins at the CN of seed ageing in rice. The results showed that the germination rate of seeds decreased by less than 20% at the CN; however, the abundance of 112 proteins and the carbonylation levels of 68 proteins markedly changed, indicating oxidative damage. The abundance and activity of mitochondrial, glycolytic, and pentose phosphate pathway proteins were reduced; consequently, this negatively affected energy production and germination. Proteins related to defense, including antioxidant system and heat shock proteins, also reduced in abundance. Overall, energy metabolism was reduced at the CN, leading to a decrease in the antioxidant capacity, whereas seed storage proteins were up-regulated and carbonylated, indicating that the seed had a lower ability to utilize seed storage proteins for germination. Thus, the significant decrease in metabolic activities at the CN might accelerate the loss of seed viability.

A notable characteristic of seed viability is the reverse S-shaped survival curve during ageing, which includes a plateau phase (Phase I; P-I), followed by a rapid decreasing phase (Phase II; P-II) and a slow decreasing phase (Phase III; P-III). The transformation from P-I to P-II is defined as the critical node (CN), which is highly important for seed conservation^1^. The average germination of approximately 42,000 diverse accessions stored for 16 to 81 years at the National Center for Genetic Resources Preservation, USA has been decreased by 42%^2^. The average germination rate of peanut (stored for 34 years), soybean (stored for 36 years), wheat (stored for 43.6 years), and barley (stored for 44.4 years) is 6%, 21%, 73%, and 86%, respectively^3^. Similar results have been also reported by the Genebank of the Leibniz Institute of Plant Genetics and Crop Plant Research (IPK), Germany^4^. Rice is extremely important food crop. One of main aims in genebanks is maintaining the rice seed safe conservation. At the T.T. Chang Genetic Resources Center in International Rice Research Institute, 183 rice accessions stored up to 30 years showed more than 70% germination^5^, and more than 93% of seed lots produced in 1980 still maintained 85% germination after 33 years in storage^6^. Owing to the reduction in seed viability, the regeneration of genetic resources is considered crucial for maintaining genetic integrity. Previous studies have shown that seed regeneration needs to be carried out prior to the CN in order to prevent a large decrease in viability, which can lead to changes in genetic composition^7^,^8^. Previously, we showed that the mitochondrial ultrastructure of seed at the CN is abnormal owing to the decreased oxygen consumption as well as the decreased activity of cytochrome *c* oxidase and malate dehydrogenase (MDH)^1^.

The role of reactive oxygen species (ROS) in the loss of seed viability has been well investigated. During natural or accelerated ageing, the levels of seed antioxidative enzymes (e.g., superoxide dismutase, SOD; catalase, CAT; ascorbate peroxidase, APX; and glutathione reductase, GR) and antioxidants (ascorbic acid and glutathione) decrease, leading to the accumulation of ROS and consequently oxidative damage^9^,^10^. The proteomic analysis of aged maize seeds indicated that the loss of seed viability loss is related to ROS damage^11^. The reduction in antioxidant capacity, i.e., decrease in the expression of *CAT1, APX1*, and *MDHAR1* may be responsible for the loss of rice seed viability during storage^12^. The mitochondrial structure and function alter in aged seeds. For instance, in aged soybean seeds, the mitochondrial ascorbic acid and glutathione cycle activity decreased, leading to elevated ROS accumulation^13^. The aged seed induces dynamic changes in mitochondrial physiology via the increased ROS production, resulting in an irreversible loss of seed viability^14^. Seed possess many repair enzymes, such as PROTEIN l-ISOASPARTYL O-METHYLTRANSFERASE, for proventing age-induced ROS accumulation to improve seed vigor and longevity^15^.

ROS accumulation can induce the formation of protein carbonyls that affect enzyme activity and lead to ageing or death^16^,^17^. Numerous studies have reported that protein carbonylation contributes to leaf and fruit senescence as well as the decreased rate of seed germination^18^,^19^,^20^. In *Arabidopsis*, HSP70 and LEA were carbonylated after ageing treatment^21^, whereas seed storage proteins (SSPs) were carbonylated during storage^22^. In this study, we aimed to determine the changes in protein abundance and protein carbonylation at the CN of seed ageing in rice. The carbonylated protein patterns were analyzed by two-dimensional (2D) gel electrophoresis followed by western blotting with antidinitrophenyl hydrazone (DNP) antibodies. The carbonylation level and pattern of several proteins might be indicators of seed ageing, and could help to improve seed storage management.

## Results

### Proteomic and carbonylation profile analysis at the CN

In our previous study, rice seed vigor loss displayed a P-I, and then experienced a rapid decreasing phase after 84% germination (P-II). Therefore, we chose the seed germination percentage at 84% as the critical node^1^. Seed vigor was analyzed from maximum to the CN, as it is this stage that is extremely important for safe conservation of seeds in genebank. This differed to previous studies in *Arabidopsis*^21^, maize^11^, and *Brassica napus* seeds^23^ where comparison was made at the end of Phase II.

**Figure 1:**
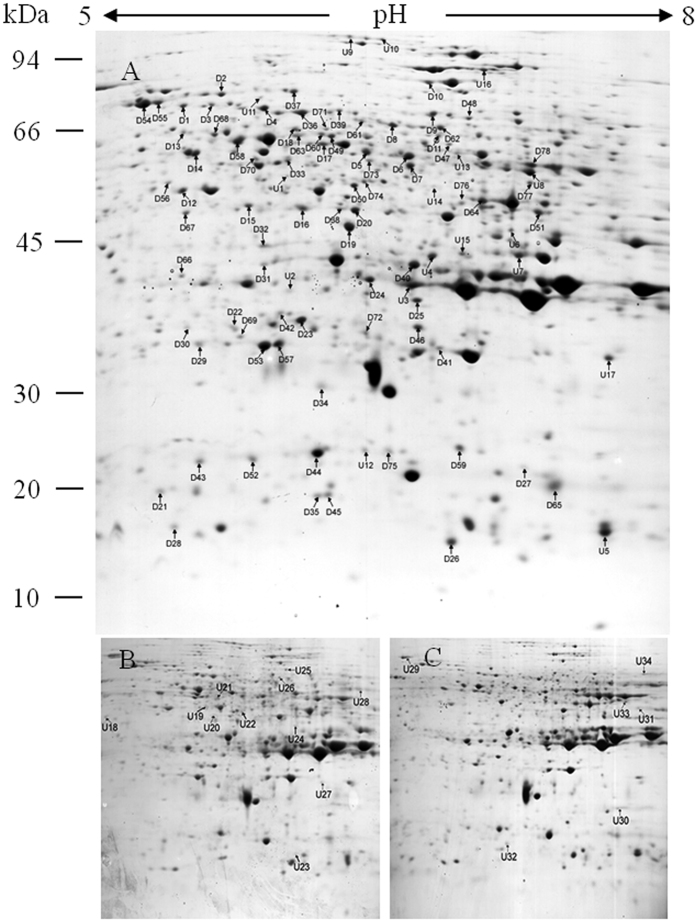
Representative isoelectric focusing (IEF)/dodecyl sulfate polyacrylamide gel electrophoresis (SDS-PAGE) separation gels of proteins from 0 d (**A**), 3 d (**B**) and 4 d (**C**) aged rice seeds after imbibition for 48 h. Total 500 μg protein were separated by immobilized pH gradient (IPG) strips and 12% (w/v) SDS-PAGE gels. Protein codes correspond to those in Tables 1, 2 and 3. Number on the left represents the apparent molecular mass. Number above the gels represents the pI of separated protein spot. U, upregulation; D, downregulation.

**Figure 2:**
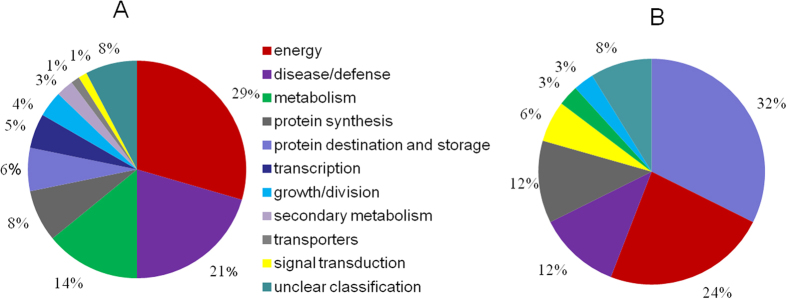
Classification of downregulated (**A**) and upregulated (**B**) proteins in 0 d, 3 d, and 4 d aged rice seeds after imbibition for 48 h.

Proteomic and carbonylation profile analysis was carried out to determine the impact of oxidative stress at the CN. Protein profiles of rice embryos was extracted from 97% (control), 92% and 84% germination percentage after 0 d, 3 d, and 4 d aged treatment, respectively, and separated by gel electrophoresis using immobilized pH gradient (IPG) strips in isoelectric focusing (IEF). Three biological repeats were used for either gels or blots of each sample. More than 700 protein spots were detected on 12% (v/v) sodium dodecyl sulfate polyacrylamide gel electrophoresis (SDS-PAGE) gels by PDQuest 8.0 (Fig. 1). The abundance of 112 protein spots showed a change higher than 1.5-fold at the CN which MOWSE score were higher than 65 (Table 1). Of them, 78 downregulated proteins (D1–D78) and 17 upregulated proteins (U1–U17) were identified in all treated seeds; 11 upregulated proteins (U18–U28) were uniquely detected in 3-d aged seeds; and six upregulated proteins (U29–U34) were uniquely detected in 4-d aged seeds (Tables 1 and 2). Figure 2 shows the change pattern of different proteins related to energy, defense, metabolism, growth or division, transcription, and other unknown functions^24^.

**Figure 3:**
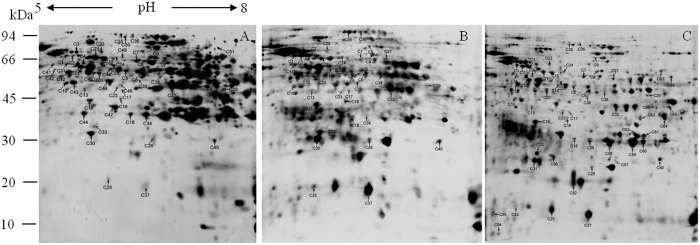
Two-dimensional (2D) immunoblots using antidinitrophenyl hydrazone antibody to detect carbonylated embryo proteins in 0-d (**A**), 3-d (**B**), and 4-d (**C**) aged rice seeds after imbibition for 48 h. Total 500 μg protein were numbered in a preparative 2D electrophoresis gel and excised for MS/MS analysis, corresponding to the proteins in Tables 1, 2 and 3. Number on the left represents the apparent molecular mass. Number above the gels represents the pI of separated protein spot. (**C**) Carbonylated spot.

To better understand protein carbonylation at the CN, we performed in-strip derivatization with 2,4-dinitrophenylhydrazine (DNPH) followed by SDS-PAGE and immumochemical detection of carbonylated proteins. Figure 3 shows carbonylated proteins from rice seeds in 2D blots. The level of carbonylated proteins on the polyvinylidene difluoride (PVDF) membrane was normalized to the protein level of the corresponding protein spot on 2D gels, and only reproducible differences were considered to be changes. The results showed that 32 (C1–C32) out of 78 downregulated proteins and 8 out of 36 upregulated proteins (C33–C40) displayed significant carbonylation. Additionally, 11 upregulated proteins (C41–C51) and 17 downregulated proteins (C52–C68) showed no significant change in abundance on 2D gels, but displayed significant changes in carbonylation (Table 3). Overall, seed proteins underwent carbonylation at the CN.

### Downregulated proteins at the CN

The 78 downregulated proteins were related to energy (29%), defense (21%), metabolism (14%), protein synthesis (8%), protein destination and storage (6%), transcription (5%), growth or division (4%), secondary metabolism (3%), transporting (1%), signal transduction (1%), and other unknown functions (2%) (Fig. 2A). Additionally, carbonylation was observed among those proteins at the CN, further suggesting that the related functions could be disrupted.

#### Proteins related to energy metabolism

**Figure 4:**
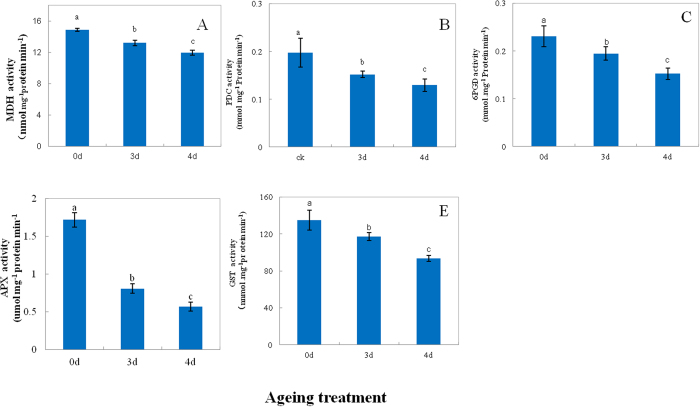
The activity of malate dehydrogenase (MDH, (**A**)) pyruvate decarboxylase (PDC, (**B**)) 6-phosphogluconate dehydrogenase (6PGD, (**C**)) ascorbate peroxidase (APX, (**D**)) and glutathione S-transferase (GST, (**E**)) in 0-d, 3-d, and 4-d aged rice seeds after imbibition for 48 h. Data represent the mean ± standard deviation of three independent experiments. All treatments significantly differed from the control at *p* < 0.05 (*n* = 3).

**Figure 5:**
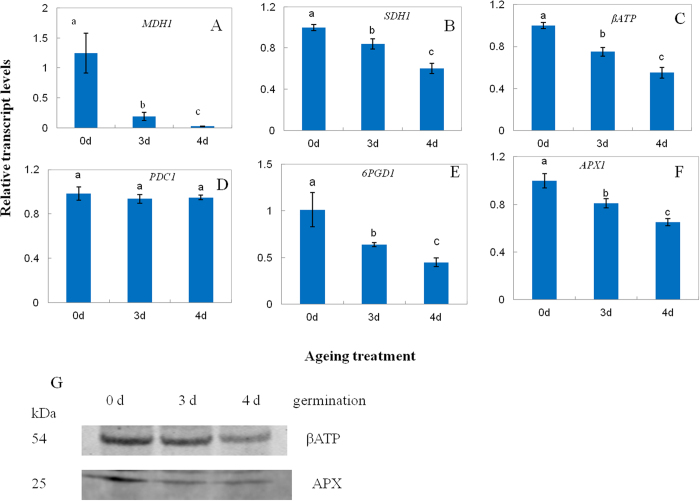
Relative levels of malate dehydrogenase 1 (*MDH1*, (**A**)) succinate dehydrogenase 1 (*SDH1*, (**B**)) βATP synthase (**C**)) pyruvate decarboxylase 1 (*PDC1*, (**D**)) 6-phosphogluconate dehydrogenase 1 (*6PGD1*, (**E**)) and ascorbate peroxidase 1 (*APX1*, (**F**)) and abundance of beta subunit of ATP synthase (βATP) and ascorbate peroxidase (APX) (**G**) in 0-d, 3-d, and 4-d aged rice seeds after imbibition for 48 h. Transcript levels in 3-d and 4-d aged seeds were calculated in relation to a value of 1.0 that assigned to 0-d aged seeds after imbibition for 48 h. Data represent the mean ± standard deviation of three independent experiments. All treatments significantly differed from the control at *p* < 0.05 (*n* = 3).

A total of 16 downregulated proteins were related to energy metabolism (Table 1). The β-ATP synthase subunit (βATP), MDH, and succinate dehydrogenase (SDH) showed decrease in abundance (D14, D19, and D48) and significant carbonylation (C12, C16, and C27) (Table 1). To better understand protein expression at the CN, the activity of MDH was measured in aged seeds after imbibition for 48 h. As compared to the control, the activity of MDH showed a decrease by 11% and 20% in 3-d and 4-d aged seeds, respectively (Fig. 4A), which was consistent with the decrease in abundance. *MDH1* also displayed a steady downregulation with ageing (Fig. 5A). Compared with the control, *SDH1* showed a decrease by 16% and 40% in 3-d and 4-d aged seeds, respectively (Fig. 5B). The immunodetection of the βATP subunit showed a significant decrease at the CN (Fig. 5G). Compared with the control, β*ATP* showed a decrease by 25% and 45% in 3-d and 4-d aged seeds, respectively (Fig. 5C). These results indicated that mitochondrial metabolism significantly decreased at the CN.

Seven proteins of the glycolytic pathway, including phospoglycerate mutase (D4), pyruvate decarboxylase (PDC, D8), triosephosphate isomerase (D29 and D53), D-glyceraldehyde 3-phosphate enolase (D58 and D63), and pyrophosphate-dependent phosphofructokinase (D62) were downregulated, indicating that glycolytic metabolism was also *reduced* at the CN. Of these proteins, D4, D8, D29, and D63 also showed significant carbonylation (C4, C7, C30 and C31) (Table 1). Compared with the control, the activity of PDC showed a decrease by 23% and 30% in 3-d and 4-d aged seeds, respectively (Fig. 4B). In this study, *PDC1* did not show any significant change at the CN (Fig. 5D). Additionally, 6-phosphogluconate dehydrogenase (6PGD, D7) of the oxidative pentose phosphate pathway (PPP) showed a decrease in abundance and significant carbonylation (C6). Compared with the control, the activity of 6PGD showed a decrease by 15% and 33% in 3-d and 4-d aged seeds, respectively, whereas *6PGD1* showed a decrease by 37% and 56%, respectively (Figs 4C and 5E). These results indicated that PPP was significantly inhibited at the CN.

#### Proteins related to defense

In this study, 12 downregulated proteins were related to defense, indicating the decreased ability of aged seeds to combat oxidative stress. Compared with the control, the activity of ascorbate peroxidase 1 (APX, D57) decreased by 47% and 33% in 3-d and 4-d aged seeds, respectively (Fig. 4D). *APX1* showed a steady decrease with ageing (Fig. 5F). Additionally, the reduced abundance in cytosolic APX proteins at the CN was confirmed by immunodetection using the cytosolic APX antibody (Fig. 5G) and indicated ROS accumulation at the CN of seed ageing. Compared with the control, the activity of glutathione S-transferase (GST, D46) decreased by 87% and 70% in 3-d and 4-d aged seeds, respectively (Fig. 4E), negatively affecting the ability of the antioxidant defense system. GST showed a decrease in abundance and significant carbonylation (C26). Five heat shock proteins (HSPs), including HSP70 (D2, D54, and D55), 17.9-kDa class I HSP (D13), 18.0-kDa class II HSP (D44), and two chaperonins (D13 and D60) significantly downregulated at the CN, whereas the HSP D2 and D44 and the chaperonin D13 showed significant carbonylation (C2, C25 and C11).

### Upregulated proteins at the CN

Compared with the control, 31 upregulated proteins were related to storage, energy, disease and defense, metabolism, protein synthesis, growth and division, and other unknown functions (Fig. 2B). SSPs formed the largest group of upregulated proteins that included globuline, glutelin, and cupin (Table 2), which showed various experimental molecular weights, indicating that SSPs were post-translationally modified or broken down (Fig. 3). In this study, we also identified 17 SSPs that displayed significant carbonylation, of which 10 were upregulated, whereas the rest showed no change in abundance (Tables 2 and 3).

## Discussion

The viability of aged seeds is an important aspect for maintaining genetic diversity during seed storage. In this study, we demonstrated the occurrence of oxidative damage that primarily affects the abundance of proteins related to energy generation, metabolism, and defense. These findings were in agreement with previous studies in *Arabidopsis*, which showed that proteins related to energy, metabolism, and defense play key roles in seed maintenance and are downregulated at the CN^21^,^22^. The changes occurring in protein abundance appeared to be related to oxidative stress^25^,^26^, resulting in an inability to utilize seed storage proteins, as the latter increased in abundance at the CN.

Proteins related to energy metabolism formed the largest group of downregulated proteins (Fig. 2A). The capacity of energy supply is highly important for seed germination and seedling growth, since large amounts of energy are needed prior to the establishment of photosynthesis in the plant^27^. Previous studies in *Arabidopsis* reported that one of the earliest events during germination is the upregulation of approximately 600 genes that encode proteins related to mitochondrial functions and are critical for germination^28^,^29^. A variety of mitochondrial mutants involved in electron transport showed slow germination rates or high seedling lethality^28^. The downregulated proteins of mitochondria were observed by carbonylated modification, including βATP, MDH and SDH, respectively (Table 1). Mitochondria plays key roles for energy supplying during seed imbibitions. βATP is the F_0_ sector of ATPase^30^. The state and function of ATP synthase machinery may determine the energy supplying. βATP protein and *βATP* expression level was significant downregualted at the CN (Fig. 5C,G). Our results were consistent with our previous reported in purified mitochondria from the CN of rice seed ageing^1^ that ATP synthase machinery was inactive which leads the ATP supplying was inhibited at the CN. The seed of MDH1 and MDH2 double knockout *Arabidopsis* mutants had a lower content of 2-oxoglutarate during imbibition^31^. In the present study, the activity of MDH and the levels of *MDH1* were significantly decreased and carbonylated in aged seeds compared with the control (Figs 4A and 5A), indicating that the TCA cycle was inhibited at the CN after seed imbibition. In addition, SDH is a key member of the electron transport chain complex II, which catalyzes the oxidation of succinate to fumarate with the reduction of ubiquinone to ubiquinol and participates in succinate dependent O_2_ consumption in the electron transport chain^32^. SDH1 knockout *Arabidopsis* mutants had a decreased activity of the electron transport chain, but an increased amount of ROS under environmental stress^33^. We showed that the level of *SDH1* was significantly decreased and carbonylated at the CN (Fig. 5B); these results were in agreement with our previous findings that succinate dependent O_2_ consumption is reduced at the CN^1^. The decreased activity of SDH might cause ROS accumulation and oxidative damage. Overall, these results demonstrated that the mitochondrial metabolism was inhibited at the CN, decreasing the production of ATP and its intermediates, which are needed for seed germination and ROS accumulation.

Several proteins whose showed a decrease in proteins abundance and exhibited different carbonylation levels were observed which involved in the glycolytic pathway, including phospoglycerate mutase which catalyzes the conversion of 3-phosphoglycerate to 2-phosphoglycerate^34^, pyruvate decarboxylase (PDC) which catalyses the decarboxylation of pyruvic acid to acetaldehyde and carbon dioxide^35^, triosephosphate isomerase which catalyzes the reversible interconversion of the triose phosphate isomers dihydroxyacetone phosphate D-glyceraldehyde 3-phosphate^36^, enolase which catalyzes the conversion of 2-phosphoglycerate to phosphoenolpyruvate^37^, and pyrophosphate-dependent phosphofructokinase which catalyzes the reversible phosphorylation of fructose-6-phosphate to fructose-1,6-bisphosphate^38^. Interestingly, PDC gene family was induced in response to environmental stress in plants^39^,^40^. By comparison, *PDC1* level showed no significant change in the aged seed (Fig. 5D). We propose that the decrease of the PDC activity (Fig. 4B) caused by carbonylation may result in deleterious downstream pathway of glycolytic metabolism, leading to a reduction of energy supplying. These results indicated that glycolytic metabolism was also reduced at the CN. 6PGD was carbonylated modification and showed downregulation in aged seed, which converts 6-phosphogluconate to ribose-5-phosphate and produces NADPH in oxidative pentose phosphate pathway (PPP)^41^. The decrease in the activity and transcript level of 6PGD resulted in reduction of NADPH production, indicating that PPP metabolism was inhibited at the CN. Taken together, the key members of energy supplying were downregulated and carbonylated modification at the CN, which might cause impairment of mitochondrial, glycolysis and PPP metabolism and then lead to decrease in energy supplying during imbibition, in turn, trigger a cascade of deleterious metabolism which could contribute to the process of ageing disorders.

Proteins related to defense were downregulated at the CN (Fig. 2A). APX1 plays an important role in the antioxidative metabolism, since it catalyzes the conversion of H_2_O_2_ to H_2_O in the ascorbate-glutathione cycle^42^. Compared with the activity of APX, the protein and gene level showed a significant decrease at the CN (Fig. 5F,G), indicating that the antioxidative system was inhibited at the CN. These results were consistent with previous findings in the aged seed of soybean, maize, and other species^11^,^12^,^13^. The decreased capacity of the antioxidative system could lead to ROS accumulation, reflecting the oxidative damage and increased protein carbonylation.

GST catalyzes the conjugation of the reduced form of glutathione to xenobiotic substrates for the purpose of detoxification^43^ and protects against oxidative damage by ROS^44^. In the present study, the activity of GST showed significant decrease at the CN (Fig. 4E), indicating that rice seed was affected by ROS. Additionally, we observed the specific carbonylation of proteins related to oxidative stress such as HSPs and chaperonins. HSPs are encoded by multigene families^45^; small HSP class I (17.9 kDa) belongs to the small HSP family, which is expressed in rice seed^45^,^46^. HSPs and chaperonins play an important role in protein folding and protect cells against stress^47^. Carbonylated HSPs and chaperonins were described in aged *Arabidopsis* seeds^22^. The downregulation of HSPs and chaperonins disrupted the defense system and might cause oxidative damage at the CN.

Our study revealed the carbonylation of upregulated proteins, such as SSP, at the CN. Previous studies reported that the degradation of storage proteins is associated with seed viability^48^ and that the seed of SSP mutants is sensitive to ageing^22^. In the present study, SSP displayed a significant upregulation and various experimental molecular weights at the CN (Figs 2B and 3). Our results suggest that the seed had a decreased ability to utilize SSPs at the CN, which might cause SSP accumulation and carbonylation. Previous studies reported that SSP might be a primary target for oxidative stress and protect other proteins for oxidative damage during seed ageing^22^,^49^. Our results suggest that the increased carbonylation level of SSP indicates the relatively high ROS levels at the CN. Our results were consistent with the decreased capacity of the antioxidant system at the CN.

## Conclusion

This study revealed that oxidative stress was related to the loss of seed viability. Our findings indicated the effects of oxidative stress at the CN, since the proteins related to energy and defense metabolism were downregulated and/or inactivated. The ability to utilize SSPs was also reduced, leading to a rapid decline in seed viability at the P-II.

## Materials and Methods

### Plant material and treatments

Rice seed (*Oryza sativa* L. *japonica nipponbare*) was obtained from the Jiangxi Agricultural Academy of Sciences, Nanchang, China. Rice seeds were treated at 40 °C and 75% relative humidity for 0 d, 3 d, and 4 d to decrease the germination rate by 3%, 8%, and 16%, respectively^1^. The embryos were extracted after seed imbibition at 28 °C for 48 h in the dark, and then stored at −80 °C until analysis.

### Proteomic and carbonylation analysis

The frozen embryos were ground with a mortar and pestle using a buffer, containing 0.1 M Tris-HCl (pH 7.5), 0.1% dithiothreitol (DTT), 2% polyvinylpolypyrrolidone (PVPP), and 0.5% ethylenediaminetetraacetic acid (EDTA). Protein was extracted using the Tris-phenol protocol^50^, and then, 500 μg of protein was applied to rehydrated gel strips with an immobilized linear pH gradient of 5–8 (BioRad, Hercules, CA, USA). The first-dimensional IEF was performed at 20 °C on a flat-bed electrophoresis unit (BioRad) as follows: rehydration for 12 h, 0 V to 150 V in 15 min, 150 V to 1,000 V in 1 h, 1,000 V to 8,000 V in 5 h, and 8,000 V until a total of 60 kVh. After IEF, the strips were: 1) equilibrated for 15 min in Solution A, containing 6 M urea, 20% (v/v) glycerol, 2% (w/v) SDS, 375 mM Tris-HCl (pH 8.8), and 2% (w/v) DTT, and then, in Solution B, containing 6 M urea, 20% (v/v) glycerol, 2% (w/v) SDS, 375 mM Tris-HCl (pH 8.8), and 2.5% iodoacetamide for analyzing the proteomic profile and 2) derivatized by incubating in 10 mM DNPH for 20 min with gentle agitation, and then, equilibrated as described above for analyzing the profile of carbonylated proteins. The equilibrated strips were placed on SDS-PAGE using homogenous 12% (v/v) polyacrylamide gels with 4% (v/v) stacking gels (BioRad). Gel electrophoresis was performed at 250 V with circulating cooling using a running buffer, containing 25 mM Tris (pH 8.3), 195 mM glycine, and 0.1% (w/v) SDS. The gels were stained with CBB G250 or transferred to PVDF membrane and detected with anti-DNP antibodies. Gel images were obtained using a flatbed scanner and analyzed by PDQuest 5.0 (BioRad). Spot intensity was calculated according to the relative expression volume. Spots with a fold change higher than 1.5 were excised for mass spectrometry.

### In-gel digestion, mass spectrometry, and database searching

The protein and carbonylated protein that were corresponded the protein spot on 2D gels, were excised into 1-mm^3^ pieces from 2D gels and de-stained with 25 mM NH_4_CO_3_ in 50% acetonitrile. The gels were dehydrated by adding acetonitrile and then, digested with 25 μl of 0.1 mM trypsin in 25 mM NH_4_CO_3_ for 15 min^51^. The excess trypsin solution was removed, and gel pieces were incubated at 37 °C overnight in 20 μl of 25 mM NH_4_CO_3_. A mixture of 1 μl peptide solution and 1 μl matrix solution with 1 mg ml^−1^, a-cyano-4-hydroxycinnamic acid in 70% acetonitrile, and 0.1% trifluoroacetic acid was loaded onto the AnchorChip MALDI target plate (Bruker Daltonics, Manning Park Billerica, MA, USA) and analyzed by a matrix assisted laser desorption-ionization time of flight (MALDI-TOF)/TOF mass spectrometer (Bruker Daltonics), according to the manufacturer’s instructions. MS data were uploaded to the Mascot server using Biotools (Bruker Daltonics) and searched against the National Center for Biotechnology Information (NCBI) protein database (25,010,123 sequences; 8,625,376,125 residues; search parameters, rice; proteolytic enzyme, trypsin; maximum missed cleavages, 1; fix modifications, carbamidomethyl; variable modifications, oxidation; peptide mass tolerance, 100 ppm; fragment mass tolerance, 0.5 Da). Spots with a Mowse score higher than 65 were considered as proteins.

### Assay of enzyme activities

The activity of MDH was determined at 340 nm as described by Glatthaar *et al*.^52^. The activity of PDC was measured in a reaction medium, containing 200 mM Tris-HCl (pH 6.0), 100 mM pyruvate, 0.1 mM thiamine pyrophosphate, 1.0 mM MgCl_2_, 9.0 units of alcohol dehydrogenase, and 225 μM NADH as described^53^ with minor modifications. The activity of APX was determined at 290 nm as described by Nakano and Asada^54^. The activity of GST was determined at 340 nm as described by Habig *et al*.^55^. The activity of 6PGD was determined in 50 mM Tris-HCl (pH 7.5) and 0.25 mM NADP, started with 2 mM 6-phosphogluconate as described by Bailey-Serres and Nguyen^56^.

### Immunoblot analysis

Equal amounts of rice embryo proteins (10 μg per lane) were loaded onto SDS-PAGE gels, transferred to PVDF, and incubated with βATP and APX antibodies. Anti-rabbit IgG was used as a secondary antibody (Agrisera, Vannas, Sweden). Immunodetection was performed using the Chemiluminescent Substrate Kit (KPL, Hemet, CA, USA).

### Quantitative real-time polymerase chain reaction (qRT-PCR)

Total RNA was isolated from rice embryos after seed imbibition for 48 h using the RNAprep pure plant kit (Tiangen, Beijing, China). Specific primer pairs (Supplemental Table 1) were designed using Primer 5 (Premier Biosoft, Palo Alto, CA, USA). qRT-PCR was performed using the Roche LightCycler^®^ 480II. The thermal cycling program was as follows^57^,^58^: 95 °C for 15 min, 40 cycles at 95 °C for 15 s and at 60 °C for 30 s. The gene expression of rice *UBQ5* was used as an internal control.

### Protein concentration assay

Protein concentration was determined as described by Bradford^59^ using bovine serum albumin as a standard.

### Statistical analysis

Data were pooled across repeated experiments. Analysis of variance in conjunction with the least significant difference test was performed by SPSS (IBM, Chicago, IL, USA). Differences were considered significant at *p* < 0.05.

## Additional Information

**How to cite this article:** Yin, G. *et al*. Proteomic and Carbonylation Profile Analysis at the Critical Node of Seed Ageing in *Oryza sativa.* *Sci. Rep.* **7**, 40611; doi: 10.1038/srep40611 (2017).

**Publisher's note:** Springer Nature remains neutral with regard to jurisdictional claims in published maps and institutional affiliations.

## Supplementary Material

Supplementary Information

## Figures and Tables

**Table 1:** Proteins with significantly decreased abundance at the critical node in 0-d, 3-d, and 4-d aged rice seeds.

Spot	Protein name	Accession No.	Scores	Fold		Carbonylation
				0d/3d	0d/4d	
**Energy**						
D1	Vacuolar proton-ATPase	NP_001058280.1	890	1.85	4.54	C1
D4	Phospoglycerate mutase	NP_001044625.1	1094	1.59	2.74	C4
D5	ATP synthase lipid-binding protein	YP_002000594.1	773	1.82	2.55	C5
D6	ATP synthase lipid-binding protein	YP_002000594.1	744	1.71	3.01	
D7	6-phosphogluconate dehydrogenase 1	NC_029261.1	1067	2.93	5.99	C6
D8	Pyruvate decarboxylase	BAC20138.1	446	1.49	2.29	C7
D14	ATP synthase subunit beta	NP_001043900.1	1482	1.59	2.11	C12
D19	Malate dehydrogenase	NP_001064860.1	903	1.35	30.02	C16
D22	Cyt-RPEase	NP_001063604.2	187	1.93	5.13	
D29	Triosephosphate isomerase	AAB63603.1	689	1.29	3.06	
D30	Carboxymethylenebutenolidase-like protein	NP_001043244.1	536	1.16	1.42	
D36	Pyruvate decarboxylase 2	NP_001049811.1	615	1.18	2,17	C21
D48	Succinate dehydrogenase flavoprotein subunit	NP_001058845.1	123	1.26	1.82	C27
D53	Triosephosphate isomerase	AAB63603.1	995	1.26	2.44	C30
D58	Enolase	AAC49173.1	1075	1.79	1.98	C31
D61	Pyruvate decarboxylase 1	NC_029260.1	147	1.19	2.39	
D62	Phosphofructokinase beta subunit	NP_001057284.1	596	1.04	2.66	
D63	Beta-enolase	AAC49173.1	269	2.34	1.48	
D64	ADH1	ADH03842.1	997	2.36	1.87	C32
D69	Vacuolar ATP synthase 16 kDa proteolipid subunit	AAO72561.1	145	∞	∞	
D70	Ketol-acid reductoisomerase	NP_001043738.1	186	∞	∞	
D74	UDP-glucose 6-dehydrogenase 3	NP_001051328.1	568	∞	∞	
D78	Glyceraldehyde-3-phosphate dehydrogenase 2,	NP_001053139.1	451	∞	∞	
**disease/defense**						
D2	70 kDa heat shock protein	ABF95267.1	502	1.51	2.33	C2
D13	Chaperonin CPN60-1, mitochondrial	NP_001048938.1	248	2.40	3.24	C11
D26	Dehydration stress-induced protein	NP_001064434.1	192	1.49	2.58	
D38	Salt tolerance protein 5	NP_001057221.1	153	1.16	4.14	C23
D44	17.9 kDa class I heat shock protein	NP_001049657.1	686	1.06	1.51	C25
D46	Glutathione S-transferase 2	NP_001044339.1	282	1.39	2.46	C26
D52	18.0 kDa class II heat shock protein	NP_001042231.1	339	1.17	1.88	
D54	70 kDa heat shock protein	ABA95501.2	813	1.49	2.55	
D55	70 kDa heat shock protein	NP_001044757.1	725	1.46	2.42	
D56	Silver leaf whitefly-induced protein 1	NP_001047794.1	653	1.38	1.50	
D57	L-ascorbate peroxidase 1	NP_001049769.1	639	3.32	10.89	
D59	Cold shock domain protein 2	NP_001060914.1	525	1.91	1.94	
D60	TCP-1/cpn60 chaperonin	AAT77033.1	338	1.28	1.82	
D72	Germin-like protein 8-2	AAC04834.1	136	∞	∞	
D73	GDP-mannose 3,5-epimerase 2	NP_001068183.1	290	∞	∞	
D75	Germin-like protein 8-2	AAC04834.1	106	∞	∞	
**Metabolism**						
D10	5-methyltetrahydropteroyltriglutamate-homocysteine methyltransferase	ABG22095.1	1087	2.89	4.92	C9
D15	Glutamine synthetase cytosolic isozyme 1-1	NP_001048045.1	328	1.88	3.78	C13
D16	Reversibly glycosylated polypeptide	CAA77235.1	178	1.63	9.04	
D17	Methylmalonate semi-aldehyde dehydrogenase	NP_001059082.1	412	2.23	4.65	C14
D18	Ketol-acid reductoisomerase	NP_001043738.1	663	1.50	4.47	C15
D20	Reversibly glycosylated polypeptide	CAA77235.1	906	1.53	2.93	C17
D37	Phosphoglucomutase	NP_001051066.1	470	1.09	6.36	C22
D41	Proteasome subunit beta type-1	NP_001063603.1	663	1.18	2.33	
D47	Inosine-5’-monophosphate dehydrogenase 1	AAK09225.1	598	1.17	1.94	
D49	Leucyl-cystinyl aminopeptidase	Q6K669.1	1325	1.37	3.83	C28
D50	S-adenosylmethionine synthase	P93438.1	638	1.61	2.21	
**protein synthesis**						
D32	60 S acidic ribosomal protein P0	NP_001060923.1	531	1.09	2.49	C19
D40	Guanine nucleotide-binding protein subunit beta	NP_001043910.1	1053	1.25	1.40	
D65	Bowman Birk trypsin inhibitor	BAD52869.1	107	1.37	1.52	
D67	Succinyl-CoA ligase [ADP-forming] subunit beta	NP_001047463.1	145	∞	∞	
D68	Mitochondrial processing peptidase beta subunit	NP_001049357.1	194	∞	∞	
D76	Mitochondrial import inner membrane translocase subunit Tim17/Tim22/Tim23 family protein	NP_001049884.1	106	∞	∞	
**protein destination and storage**						
D28	Cupin family protein	AAS07324.1	394	1.66	2.97	
D42	Cupin family protein	ABF95817.1	202	1.21	2.92	
D43	Cupin family protein	ABF95817.1	376	1.31	1.68	
D71	Cupin family protein	ABF95817.1	236	∞	∞	
D77	Cupin family protein	ABF95817.1	144	∞	∞	
**Transcription**						
D21	Elicitor-inducible protein EIG-J7	NP_001048145.1	232	1.52	5.34	
D35	Glycine-rich RNA-binding protein 7	AAT85299.1	464	1.29	3.72	
D39	Asparagine-tRNA ligase	NP_001043066.1	460	1.14	2.74	C24
D45	Glycine-rich RNA-binding protein GRP1A	NP_001067344.1	64	1.49	1.66	
**growth/division**						
D12	Actin	BAB63635.1	700	1.43	3.82	C10
D51	Late embryogenesis abundant protein 1	A2XG55.2	355	1.21	1.35	C29
D66	Spermidine synthase 1	NP_001059438.1	183	∞	∞	
**secondary metabolism**						
D31	NADH-dependent enoyl-ACP reductase	NP_001061557.1	570	1.31	6.18	
D34	Lactoylglutathione lyase	NP_001055113.1	186	1.14	4.01	
Transporters						
D9	ECF transporter A component EcfA	BAD11555.1	777	1.72	3.95	C8
**signal transduction**						
D33	GDP dissociation inhibitor	NP_001055566.1	550	1.71	2.61	C20
**unclear classification**						
D3	Os09g0491772 protein	NP_001175918.1	241	1.54	2.91	C3
D11	OSJNBa0010H02.6 protein	NP_001053500.1	748	1.67	3.05	
D23	Uncharacterized protein	NP_001056364.1	803	1.32	1.90	
D24	OSJNBa0004N05.4 protein	CAE03380.1	608	1.42	2.44	C18
D25	Uncharacterized protein P0435H01.4	NP_001044131.1	654	1.53	2.07	
D27	Uncharacterized protein P0036D10.5	NP_001174164.1	238	1.46	2.57	

Mascot scores >65 are statistically significant at *p* < 0.05.

**Table 2:** Proteins with significantly increased abundance at the critical node in 0-d, 3-d, and 4-d aged rice seeds.

Spot	Protein name	Accession No.	Scores	Fold		Carbonylation
				3d/0d	4d/0d	
**protein destination and storage**						
U14	Glutelin type-A 2	BAC77349.1	362	1.41	2.20	C39
U3	Cupin family protein	NP_001051533.1	554	1.01	2.35	C34
U4	Cupin family protein	NP_001051533.1	438	1.74	4.09	
U15	Globulin-like protein	AAM33459.2	472	1.63	1.67	
U5	Glutelin	NP_001046769.1	205	1.32	3.22	
U11	Glutelin type-A 2	CAA38211.1	274	1.83	2.81	
U8	Vicilin storage protein	AAM33459.2	479	2.42	4.04	
U18	Cupin family protein	NP_001051533.1	511	∞	0	
U27	Cupin family protein	NP_001173574.1	198	∞	0	
U28	Cupin family protein	BAC77349.1	425	∞	0	
U32	Globulin-like protein	AAM33459.2	371	0	∞	
**energy**						
U1	Pullulanase	ACY56106.1	161	1.95	3.18	
U9	H0806H05.4 protein	CAC09471.2	542	1.42	2.62	C35
U10	H0806H05.4 protein	CAC09471.2	312	3.31	5.48	C36
U12	1,4-alpha-glucan-branching enzyme	BAA01584.1	115	1.14	1.98	C37
U21	ATP-citrate synthase alpha chain protein	NP_001067052.1	196	∞	0	
U29	Peroxiredoxin-2C	NP_001043845.1	276	0	∞	
U30	Peroxiredoxin-2C	NP_001043845.1	509	0	∞	
U34	Nucleoside diphosphate kinase	NP_001065404.1	246	0	∞	
**disease/defense**						
U2	Heat shock protein 90-1	BAD04054.1	205	1.56	2.16	C33
U6	ABA-responsive protein	ABA98234.1	156	1.68	3.26	
U22	GDP-mannose 3,5-epimerase 2	NP_001068183.1	195	∞	0	
U23	Dehydration stress-induced protein	NP_001057177.1	170	∞	0	
**signal transduction**						
U13	Adenosine kinase	BAC02723.1	169	1.39	1.79	C38
U17	GTP-binding nuclear protein Ran-2	NP_001056390.1	553	2.06	2.86	C40
**metabolism**						
U16	Sucrose synthase 1	NP_001050319.1	103	2.21	3.56	
**growth/division**						
U19	Meiotic recombination protein SPO11-1	NP_001067760.1	101	∞	0	
**Unclear classification**						
U7	Os03g0327600 protein	NP_001049995.1	470	1.60	2.16	
U20	OSJNBa0044M19.9 protein	NP_001052622.1	150	∞	0	
U31	Os05g0468800 protein	NP_001055802.2	409	0	∞	

Mascot scores >65 are statistically significant at *p* < 0.05.

**Table 3:** Proteins with no significantly changed abundance and significant carbonylation at the critical node in 0-d, 3-d, and 4-d aged rice seeds.

Spot	Protein name	Accession No.	Scores
Down-regualation			
Energy			
C42	Vacuolar ATPase B subunit	NP_001057902.1	316
C46	Phosphoglycerate kinase	ABI74567.1	899
C47	Glucose and ribitol dehydrogenase homolog	Q75KH3.2	664
Transcription			
C45	Eukaryotic initiation factor 4A-1	BAA02152.1	448
C48	Elongation factor Tu	NP_001051912.1	415
Metabolism			
C50	Aconitate hydratase, cytoplasmic	Q6YZX6.1	671
C51	Pyruvate kinase 1, cytosolic	NP_001065749.1	194
Disease and defense			
C41	60 kDa chaperonin alpha subunit	AAP44754.1	215
Secondary metabolism			
C44	Lactoylglutathione lyase	BAB71741.1	797
Growth/division			
C43	Actin-1	NP_001051086.1	573
Protein destination and storage			
C49	T complex protein	NP_001057876.1	227
Up-regualation			
Protein destination and storage			
C52	19 kDa globulin	CAA45400.1	161
C53	Cupin family protein	AAS07324.1	155
C56	Glutelin type-A 3	CAA38211.1	342
C57	Glutelin type-A 1	AAA33906.1	228
C58	Glutelin type-A 2	BAA00462.1	315
C60	Glutelin type-B 5	BAC77349.1	280
C67	Cupin family protein	AAS07324.1	575
Metabolism			
C55	Proteasome subunit alpha type-6	NP_001049162.1	133
C59	Glyceraldehyde-3-phosphate dehydrogenase 2, cytosolic	NP_001053139.1	344
Energy			
C61	Formate dehydrogenase 1, mitochondrial	BAA77337.1	295
C65	Pyrophosphate-dependent phosphofructokinase alpha subuni	NP_001061602.1	263
C66	Glucose-6-phosphate isomerase, cytosolic A	BAA08148.1	178
C68	ATP synthase subunit c, chloroplastic	CAA48649.1	179
Unclear classification			
C54	Os05g0569500 protein	NP_001056364.1	361

Mascot scores >65 are statistically significant at *p* < 0.05.
