# Supplementary material for: Proteomic and Carbonylation Profile Analysis at the Critical Node of Seed Ageing in Oryza sativa
Source: Sci Rep. 2017 Jan 17;7:40611. doi: 10.1038/srep40611 (PMC5240128; doi:10.1038/srep40611)
Supplement: Supplementary Information [file srep40611-s1.pdf]

Proteomic and Carbonylation Profile Analysis at the Critical Node of Seed Ageing in *Oryza sativa*

Guangkun Yin[1+], Xia Xin[1+], Shenzao Fu[1,3+], Mengni An[1], Shuhua Wu[1], Xiaoling Chen[1], Jinmei Zhang[1], Juanjuan He[1], James Whelan[2]*, Xinxiong Lu[1]*

[1]Current Address: National Genebank, Institute of Crop Science, Chinese Academy of Agricultural Sciences, Beijing 100081, China

[2]Australian Research Council Centre of Excellence in Plant Energy Biology, School of Life Science, La Trobe University, Bundoora, Victoria 3083, Australia

[3] China National Rice Research Institute, Hangzhou 310006, China

*Corresponding authors: E-mail: luxinxiong@caas.cn; J.Whelan@LaTrobe.edu.au.

[+] These authors contributed equally to this work.

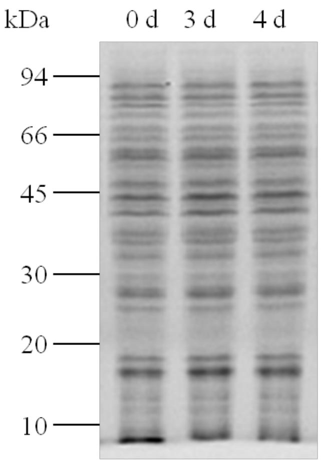

Figure S1. SDS-PAGE analysis of protein from 0 d, 3 d and 4 d aged rice seeds after imbibition for 48 h. Total 10 μg protein was separated by 12% sodium dodecyl sulfate polyacrylamide gel electrophoresis.

S1 table. List of genes used in real-time PCR.

Gene name	Accession No.	Gene description
MDH1	NP_001064860.1	malate dehydrogenase 1
SDH1	NP_001058845.1	Succinate dehydrogenase [ubiquinone] flavoprotein subunit, mitochondrial
βATP	NP_001043900.1	ATP synthase, H^+ transporting, mitochondrial F_1 complex, beta polypeptide
PDC1	NC_029260.1	Pyruvate decarboxylase 1
6PGD1	NC_029261.1	6-phosphogluconate dehydrogenase 1
APX1	NP_001049769.1	L-ascorbate peroxidase 1
UBQ5	AK062354	Ubiquitin 5
